# Defect-mediated colloidal interactions in a nematic-phase discotic solvent†

**DOI:** 10.1039/c9ra05377h

**Published:** 2019-10-17

**Authors:** Aurora D. González-Martínez, Marco A. Chávez-Rojo, Edward J. Sambriski, José A. Moreno-Razo

**Affiliations:** Departamento de Física, Universidad Autónoma Metropolitana-Iztapalapa Avenida San Rafael Atlixco No. 186, Colonia Vicentina, Delegación Iztapalapa Mexico City 09340 Mexico jamr.uam@gmail.com; Facultad de Ciencias Químicas, Universidad Autónoma de Chihuahua Circuito Universitario #1 s/n, Nuevo Campus Universitario Chihuahua Chihuahua 31000 Mexico; Department of Chemistry, Delaware Valley University Doylestown Pennsylvania 18901 USA Edward.Sambriski@delval.edu

## Abstract

Interactions between colloidal inclusions dispersed in a nematic discotic liquid-crystalline solvent were investigated for different solute–solvent coupling conditions. The solvent was treated at the level of Gay–Berne discogens. Colloidal inclusions were coupled to the solvent with a generalized sphere-ellipsoid interaction potential. Energy strengths were varied to promote either homeotropic or planar mesogenic anchoring. Colloid–colloid interactions were modeled using a soft, excluded-volume contribution. Single-colloid and colloid-pair samples were evolved with Molecular Dynamics simulations. Equilibrium trajectories were used to characterize structural and dynamical properties of topological defects arising in the mesomorphic phase due to colloidal inclusions. Boojums were observed with planar anchoring, whereas Saturn rings were obtained with homeotropic anchoring. The manner in which these topological defects drive colloidal interactions was assessed through a free energy analysis, taking into account the relative orientation between a colloidal dyad and the nematic-field director. The dynamical behavior of defects was qualitatively surveyed from equilibrium trajectories borne from computer simulations.

## Abstract 2

Saturn ring dyads (left) mediate more stable colloidal interactions than Saturn ring triads (right) in nematic-phase discotic dispersions.

## Graphical abstract

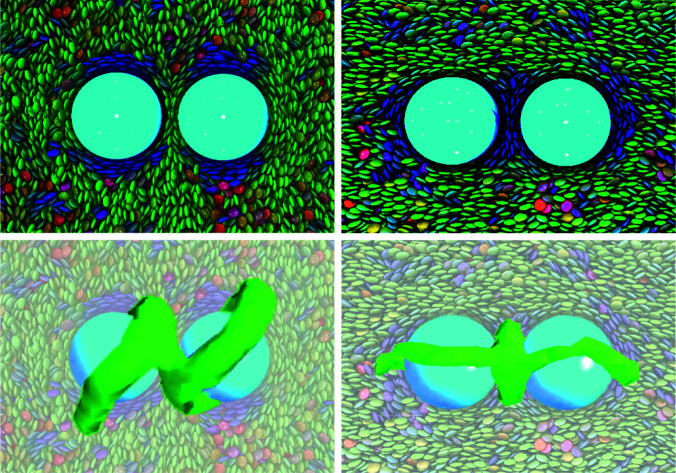

## 1 Introduction

Nematic liquid crystals with colloidal inclusions provide an avenue for producing multifunctional, tunable materials. The formation of colloidal structures can be controlled through the alignment and colloid-surface anchoring of the host liquid-crystal (LC) solvent.^1–5^ Topological defects arise when colloidal inclusions disrupt the prevalent order in the LC solvent. When coupled to the inclusions, these topological defects prompt an elastic binding interaction between guest colloidal particles.^6–10^ Defects themselves are key in designing specific colloidal arrangements and in contributing to their stability. Previous studies^11–14^ show that these non-covalent binding interactions can be sufficiently strong and capable of withstanding thermal fluctuations, of 
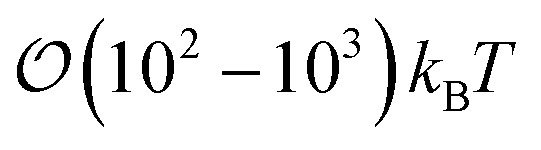
.

A mounting body of work involving host LC solvents and guest colloidal inclusions has focused on calamitic (*i.e.*, prolate) systems in 2D^12^ and 3D.^15,16^ The production of sheets,^17,18^ wires,^7,15^ glasses,^19^ crystallites,^20^ and birefringent soft solids^21^ from bulk dispersions has already been shown. Sensor-based technologies are also possible with colloid–LC composites, for instance, to probe ions,^22^ point mutations in DNA,^23^ and biochemical analytes.^24,25^ An alternative approach to the assembly of colloidal particles by LC topological defects involves trapping the inclusions at an interface. Specifically, colloidal structures can be controlled through interfacial disclinations from the LC solvent in contact with an isotropic medium (*e.g.*, air^26–28^ or oil^29^).

Discotic (*i.e.*, oblate) liquid crystals with colloidal inclusions have only been recently reviewed^30,31^ though this vein of work began nearly two decades ago. Special consideration has been given to experimental discotic-colloid systems, with an eye to the underlying mesophases and their connection to optical, dielectric, and thermodynamic properties.^32–36^ Unlike their calamitic counterparts, discotic systems have not received as much attention in the realm of solvent-assisted colloidal assembly. Research perspectives have focused, instead, on the manner in which nanoparticles enhance overall discotic systems, such as by extending the range of working conditions.^30,31^ In fact, discotic systems typically require higher temperatures to achieve the nematic (*N*_D_) phase when compared to the analogous calamitic mesophase.^37^ However, the need for lower temperatures in practical applications has been previously established.^38–40^ A recent experimental breakthrough made it possible to achieve the nematic phase in a discotic system, at room temperature, with gold nanoparticles.^41^ A handful of studies have also focused on phase transitions,^42–52^ kinetic behavior,^48^ segregation,^53–55^ and sedimentation^56^ of colloid-discotic mixtures.

Nematic LC fluids are complex, spatially-oriented systems. Mesogenic domains of LC molecules in an ensemble will align collectively in an average direction known as the director. In the presence of colloidal inclusions, however, an attractive colloid–mesogen interaction can drive an anchoring effect: LC molecules “latch on” to the colloidal surface and undergo a change in orientation with respect to the bulk-field director. Physically, the curvature of colloidal particles coupled with the anisotropic shape of the mesogenic units prevents the LC solvent from occupying volume uniformly near the spatial domain of colloids. The reorientation of LC molecules near the colloid gives rise to fluid regions similar to dislocations, causing defects and elastic distortions.^6–8^ As a result, the nematic phase displays a break in symmetry manifested by point or loop defects. Systems can be optimized so that defects “engage” with one another, giving rise to defect-mediated colloidal structures.^57^ The range of possible structures of such LC–colloid composites^58,59^ can be expanded with chiral systems.^60–63^

Surfactants and thin films^11,64^ have been used to condition colloidal surfaces, with the ability to tailor both strength and type of anchoring. In a broad sense, colloidal surfaces can be chemically functionalized to favor either homeotropic (radial) or planar (tangential) anchoring. When planar anchoring is favored, the nematic-field director of the LC medium is expected to be tangential to the colloidal surface, exhibiting a pair of antipodal defects known as boojums that align with the far-field director. On the other hand, when homeotropic anchoring is favored, a loop defect emerges known as a Saturn ring. Another level of control becomes possible with Saturn rings because they can be manipulated with optical tweezers. Additionally, Saturn rings from different colloidal particles can couple to form topological knots,^65^ which obey specific charge conservation laws.^12^ In this manner, the observed colloidal structures result from a complex set of interactions prompted by solute–solvent coupling.

Colloidal inclusions cause a (local) distortion with respect to the far-field director, which results in an energy cost taken on by the system. When several inclusions coexist, the system will tend to reduce the volume accessible to these distortions, thus minimizing the cost in solvent elastic energy. This feat is met by restricting colloidal particles to “share” defect zones and makes for the driving force behind colloidal assembly in LC solvents. In calamitic systems, stable aggregates have been obtained with interesting optical features.^66,67^ Certain properties have been linked to the resulting symmetries of the assembly, with behaviors analogous to those of photonic crystals^12,68^ and with structures consistent with biological systems.^69–71^ Our primary goal is to characterize the effective interactions mediated by topological defects in discotic LC solvents with colloidal inclusions.

The present work extends the literature with a Molecular Dynamics (MD) computer simulation study on a discotic system, as a counterpart to the information available for calamitic systems. For example, calamitic systems have been treated along this vein experimentally,^72^ theoretically,^73–76^ and with computer simulations.^77–80^ An equivalent effort is not currently available, to our knowledge, for discotic systems.^2^ The intent is to draw parallels to and highlight differences on the way topological defects drive colloidal association. In the initial effort reported here, single-colloid and colloid-pair samples are considered as “building blocks” for complex structures.^81^

In our model, the “host” solvent is a Gay–Berne discogen, with a parameter set which approximates a triphenylene core. The “guest” colloidal particles are soft spheres. Single-colloid samples are used as a reference system to compare colloid-pair samples. To ensure that attractive interactions in the system are solely due to solute–solvent coupling, and thus to better characterize the emergent interactions, the colloid–colloid potential only takes into account the excluded volume (*i.e.*, it is a soft, purely repulsive interaction). Planar and homeotropic anchoring are compared to a reference arrangement, in which neither type of anchoring is favored. In the spirit of previous studies that considered the relative orientation of colloidal pairs with the far-field director,^82,83^ we considered two limiting cases here: when the center-to-center, intercolloidal vector is initialized to be parallel (the PARA case) or perpendicular (the PERP case) to the director. In this manner, four characteristic cases arise: the manner in which topological defects interact is compared between planar (P-PARA and P-PERP) and homeotropic (H-PARA and H-PERP) anchoring. Simulation snapshots and trajectories complement our quantitative results. The treatment of a discotic model, as done in the present work, offers another avenue for structured materials by drawing on the properties of discotic systems.^84^

Our report is structured as follows: in Section 2, the model is reviewed. This includes a brief treatment of the interactions between Gay–Berne discogens, colloidal particles, and the coupling of the two species. Details on the implementation of the model in computer simulations are summarized in Section 3. This section also includes an overview of the computational details for the local order parameter and system free energy. In Section 4, results from computer simulations are presented. Findings are categorized by single-colloid and colloid-pair systems. We qualitatively review the dynamical behavior of topological defects in the four characteristic cases (*i.e.*, P-PARA, P-PERP, H-PARA and H-PERP). The relative stability of the different scenarios is also reviewed in the context of free energy. Closing remarks with a focus on defect-mediated assembly are provided in Section 5.

## 2 Model

The LC–colloid system contains *N*_c_ solid colloidal particles immersed in a nematic LC comprised of *N*_m_ mesogens. Position vectors for all colloids are denoted by {**R**} = **R**_1_,**R**_2_,…,**R**_*N*_c__, whereas for mesogens making up the solvent, position vectors are denoted by {**r**} = **r**_1_,**r**_2_,…,**r**_*N*_m__ with their respective (unit) orientation vectors by {**ê**} = **ê**_1_,**ê**_2_,…,**ê**_*N*_m__.^85^ The total interaction energy is given by the sum of three contributions,

1 *U*_tot_({**r**},{**ê**},{**R**}) = *U*_mm_({**r**},{**ê**}) + *U*_mc_({**r**},{**ê**},{**R**}) + *U*_cc_({**R**}),

where *U*_mm_({**r**},{**ê**}) is the mesogen–mesogen interaction (dependent on the positions and orientations of all mesogens), *U*_mc_({**r**},{**ê**},{**R**}) is the mesogen–colloid interaction (dependent on the positions and orientations of all mesogens, as well as the positions of all colloidal particles), and *U*_cc_({**R**}) is the colloid–colloid interaction (dependent only on the positions of all colloidal particles). The last term in eqn (1) is nonzero only when *N*_c_ ≥ 2. In our study, we considered LC–colloid systems with *N*_c_ = 1 (single-colloid systems) as well as with *N*_c_ = 2 (colloid-pair systems). All terms in *U*_tot_({**r**},{**ê**},{**R**}) are detailed in the following subsections.

### 2.1 Mesogen–mesogen interaction

The mesogen–mesogen contribution *U*_mm_({**r**},{**ê**}) assumes pairwise additive interactions. The medium consists of non-spherical, mesogenic particles interacting *via* the Gay–Berne interaction potential.^86^ An essential feature in tracking thermal effects in the system is the inclusion of both attractive and repulsive interactions, which are captured in the Gay–Berne potential. Each mesogen is represented by an oblate ellipsoid, given that the LC medium is comprised of discotic mesogens (*i.e.*, discogens). The orientation vector for each discogen is defined perpendicular to the plane containing the major axis of the ellipsoid. Because *U*_mm_({**r**},{**ê**}) only depends on the relative separation of any two mesogens *i* and *j*, the definition is specialized so that2
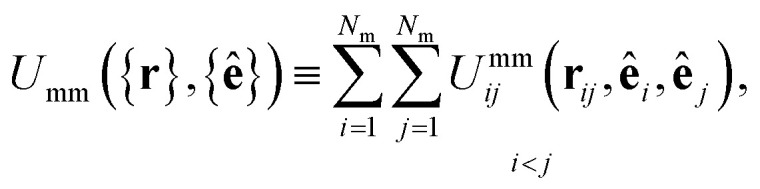
where **r**_*ij*_ = **r**_*i*_ − **r**_*j*_ is the center-to-center separation vector between the *i*th and *j*th discogen, while **ê**_*i*_ and **ê**_*j*_ are their respective unit orientation vectors. The operational form of the potential is given by

3 *U*^mm^_*ij*_(**r**_*ij*_,**ê**_*i*_,**ê**_*j*_) = 4*ε*^mm^_*ij*_([*Ξ*^mm^_*ij*_]^−12^ − [*Ξ*^mm^_*ij*_]^−6^).

The relative orientation of mesogens within the medium is taken into account through *Ξ*^mm^_*ij*_ and *ε*^mm^_*ij*_, giving rise to anisotropic intermolecular interactions. To define these factors, a fully specified function of a general variable *ω* is introduced,4
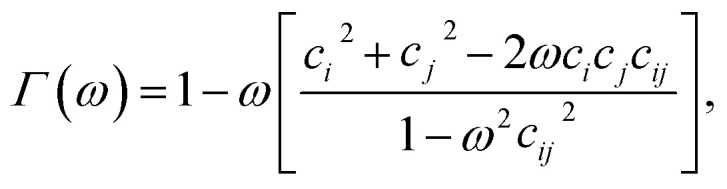
where *c*_*i*_ = **ê**_*i*_⋅**r̂**_*ij*_, *c*_*j*_ = **ê**_*j*_⋅**r̂**_*ij*_, *c*_*ij*_ = **ê**_*i*_⋅**ê**_*j*_, and **r̂**_*ij*_ = **r**_*ij*_/|**r**_*ij*_| is the unit (center-to-center) separation vector. Now,5
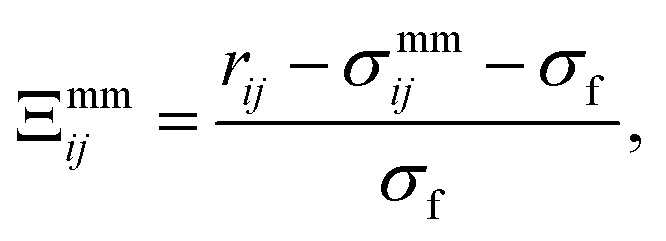
where *r*_*ij*_ = |**r**_*ij*_| is the magnitude of the separation vector, *σ*_f_ is the discogen thickness, and

**Fig. 1:**
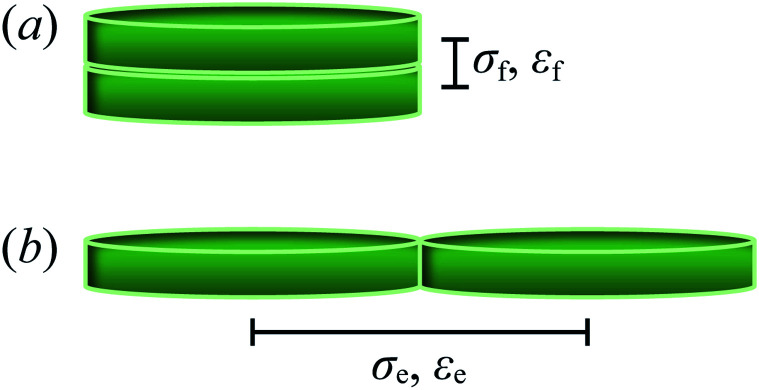
Definitions of (a) face-to-face parameters {*σ*_f_, *ε*_f_}, and (b) edge-to-edge parameters {*σ*_e_, *ε*_e_}, associated with the discogen model. Discogens are schematically represented with cylindrical geometry for simplicity, though they are actually oblate ellipsoids in the model.

6 *σ*^mm^_*ij*_ = *σ*_e_[*Γ*(*ω* = *χ*)]^−1/2^,

with7
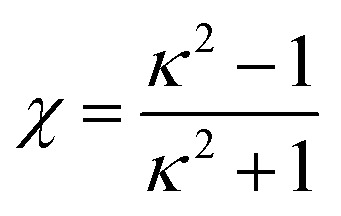
being the length anisotropy of discogens, where *κ* = *σ*_f_/*σ*_e_ is the aspect ratio between the thickness of the discogen and *σ*_e_ denotes its diameter. The designations “f” and “e” are mnemonic for face-to-face and edge-to-edge configurations, respectively, as defined in Fig. 1. For discotic mesogens, the aspect ratio is such that *σ*_f_ < *σ*_e_ and for which we have set *σ*_e_ = 1.0.

The energy scale is defined by

8 *ε*^mm^_*ij*_ = *ε*_0_[*ε*_1_(**ê**_*i*_,**ê**_*j*_)]^*ν*^[*ε*_2_(**r̂**_*ij*_,**ê**_*i*_,**ê**_*j*_)]^*μ*^,

where *ε*_0_ is the potential energy well depth for two discogens orthogonal to one another (*i.e.*, cross configuration) and to the center-to-center vector (*i.e.*, *c*_*i*_ = *c*_*j*_ = *c*_*ij*_ = 0). Moreover, *ν* and *μ* control the contribution of the two dimensionless energy factors, *ε*_1_(**ê**_*i*_,**ê**_*j*_) and *ε*_2_(**r̂**_*ij*_,**ê**_*i*_,**ê**_*j*_). In particular,

9 *ε*_1_(**ê**_*i*_,**ê**_*j*_) = [1 − *χ*^2^*c*_*ij*_^2^]^−1/2^,

controls the parallel alignment of discogens and the emergence of mesogenic phases. The second term is given by

10 *ε*_2_(**r̂**_*ij*_,**ê**_*i*_,**ê**_*j*_) = *Γ*(*ω* = *χ*′),

where11
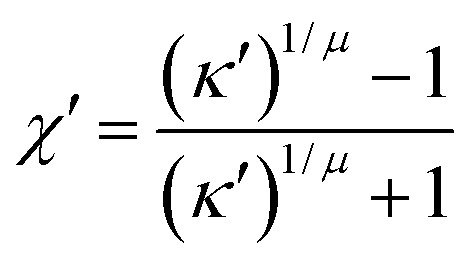
is the energy anisotropy of the discogen, with *κ*′ = *ε*_e_/*ε*_f_ being the ratio of attractive basins of the interaction energy between a face-to-face (*ε*_f_) and an edge-to-edge (*ε*_e_) configuration.

To convey different mesogenic models with the Gay–Berne potential, Bates and Luckhurst^87^ proposed the notation GB(*κ*, *κ*′, *μ*, *ν*), which is used here: the discogen parameterization in this work is GB(0.345, 0.2, 1.0, 2.0), which corresponds to a (coarse-grained) triphenylene core.^88,89^ It has been previously noted^90,91^ that *κ* = 0.345 is higher than triphenylene systems of practical importance due to neglected pendant groups from the discogenic core. Although peripheral groups on the core have been shown in the laboratory to be essential in accessing certain mesophases^84,92–96^ (particularly the *N*_D_ [discotic nematic] phase), incorporating such detail through a smaller *κ* in the coarse-grained representation only fine-tunes certain regions of the phase diagram,^89^ without forgoing the essential physics or application features in our studies. The value of *κ*′ for the current parameterization favors face-to-face over edge-to-edge configurations: this arrangement promotes the nematization of the LC at reasonable thermodynamic state points, representing a key feature in the model for this work. Phase diagrams for GB(0.345, 0.2, 1.0, 2.0) have been previously reported.^97,98^

### 2.2 Mesogen–colloid interaction

The mesogen–colloid interaction is key in the phenomenology of nematic colloid systems. A coupling of length- and timescales effectively occurs, which leads to interesting behaviors behind the break in symmetry of the solvent. The mesogen–colloid interaction depends on the orientation of the interacting mesogens and their relative position with respect to the colloids. In this work, this cross-species interaction is accounted for by12
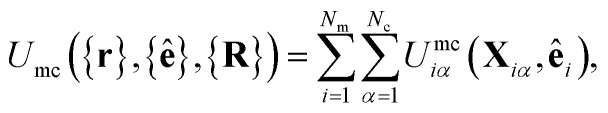
where **X**_*iα*_ is the separation vector between the *i*th discogen and the *α*th colloid (*i.e.*, **X**_*iα*_ = **r**_*i*_ − **R**_*α*_). Note the separate limits on the summations and the inclusion of the orientation vector **ê**_*i*_ for the *i*th mesogen.

The mesogen–colloid interaction is captured by a model proposed previously,^99^ which is a generalized potential between spherical and non-spherical particles. This interaction is operationally defined as13
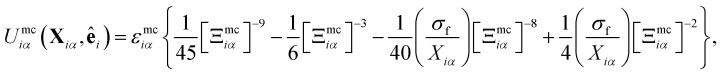
with14
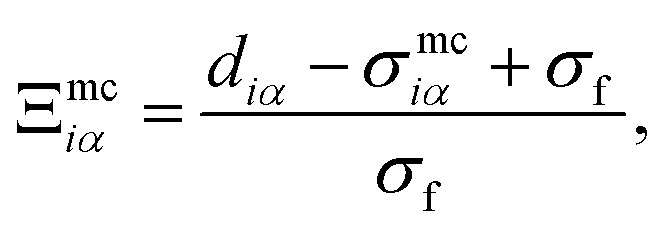
in which *d*_*iα*_ represents the separation between the discogen and the colloid surface (*i.e.*, *d*_*iα*_ = ***X***_*iα*_ − *σ*_c_/2, where ***X***_*iα*_ = |**X**_*iα*_| is the magnitude of the mesogen–colloid center-to-center separation vector and *σ*_c_ denotes the colloid diameter). Anisotropic contributions are captured through *σ*^mc^_*iα*_ and *ε*^mc^_*iα*_. Specifically,15
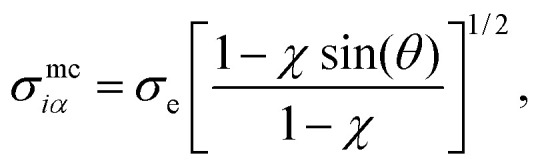
where

16 sin(*θ*) = [1 − cos^2^(*θ*)]^1/2^ = [1 − *c*_*i*_^2^]^1/2^,

with *c*_*i*_ defined as in eqn (4), but **X̂**_*iα*_ (the analogous unit vector for **r̂**_*ij*_) defined using the mesogen–colloid center-to-center separation vector and *χ* taken from eqn (7). Additionally, *σ*_e_ is the discogen diameter defined in the context of eqn (7). Now,17
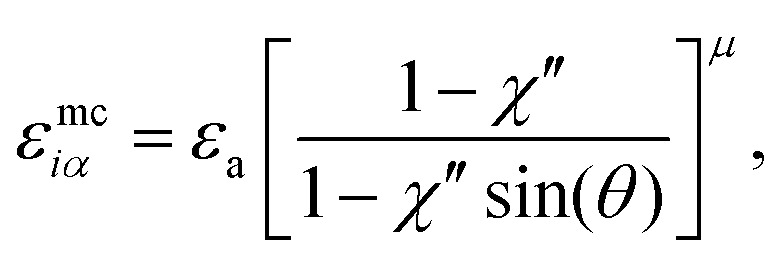
where *ε*_a_ is the LC–colloid anchoring strength (*i.e.*, an energy scale), *μ* is matched to that of eqn (8), and

**Fig. 2:**
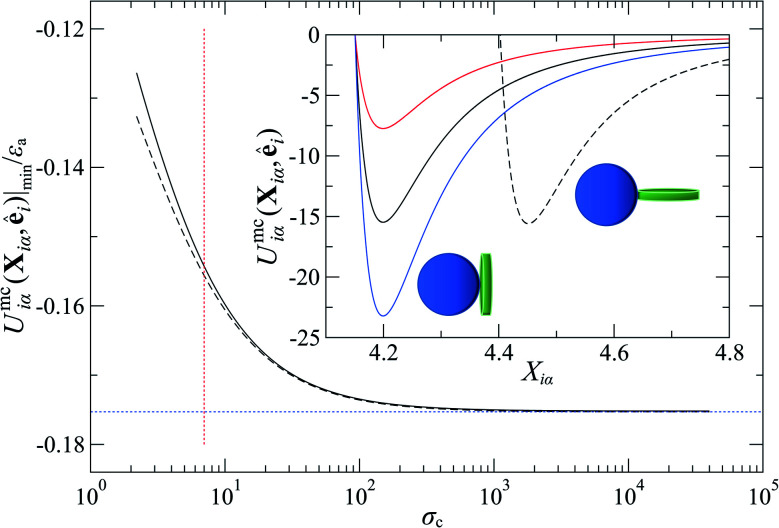
The mathematical form of *U*^mc^_*ij*_(**X**_*iα*_,**ê**_*i*_) leads to equal well depths for the LC–colloid limiting configurations shown in the inset only if the colloid diameter 
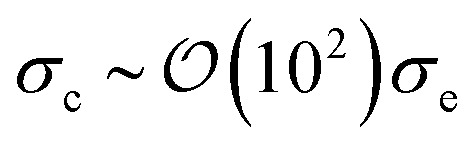
. This scenario is relevant when neither planar nor homeotropic anchoring is preferred (*i.e.*, *κ*′′ = 1). This is confirmed by tracking the minimum of *U*^mc^_*ij*_(**X**_*iα*_,**ê**_*i*_) normalized by the energy scale *ε*_a_ plotted against *σ*_c_, considering the two limiting anchoring modes [planar (dashed line) and homeotropic (solid line)]. For *σ*_c_ = 7*σ*_e_ as used in this study, well-depth equivalence for *κ*′′ = 1 is only approximate (dashed red line). The inset shows *U*^mc^_*ij*_(**X**_*iα*_,**ê**_*i*_) as a function of interparticle separation **X**_*iα*_ corresponding to data in the main plot, but specialized to *σ*_c_ = 7*σ*_e_ and *ε*_a_ = 100*ε*_0_. Other anisotropies in anchoring strengths are also shown: *κ*′′ = 0.5 [red line] and *κ*′′ = 1.5 [blue line]. Discogens are schematically represented with cylindrical geometry for simplicity, though they are actually oblate ellipsoids in the model. The relative size of colloids and discogens in the schematic are not to scale, but were chosen to emphasize distinct anchoring modes.

18 *χ*′′ = 1 − (*κ*′′)^1/*μ*^

takes into account the anisotropic contribution in *ε*_a_. In addition, *κ*′′ = *ε*_f_/*ε*_e_ adjusts the ratio of anchoring energy scales on the colloid surface, which effectively controls the anchoring mode: if *κ*′′ > 1, homeotropic anchoring is favored; if *κ*′′ < 1, planar anchoring is favored. When *κ*′′ = 1, planar and homeotropic anchoring are approximately equally favored, as shown in Fig. 2.

To explore the effects of different anchoring strength ratios, we chose *κ*′′ = {0.1, 0.2, 0.4, 0.6, 0.8, 1.0, 2.0, 5.0, 10.0} for single-colloid systems; the same values were considered for colloid-pair systems with the exception that *κ*′′ = {4.0, 6.0} was substituted for *κ*′′ = 5.0 in the set. Additionally, we fixed *ε*_a_ = 100*ε*_0_ and *σ*_c_ = 7*σ*_e_ (*i.e.*, *ε*_0_ = 1.0 and *σ*_e_ = 1.0, as indicated in Section 2.1), and *μ* = 1.0 for all samples.

### 2.3 Colloid–colloid interaction

The colloid–colloid interaction *U*_cc_({**R**}) is modeled through a soft-sphere interaction potential, which only accounts for interparticle repulsions on close approach. As such, this term only depends on the separation between colloids and is thus an isotropic contribution defined as19
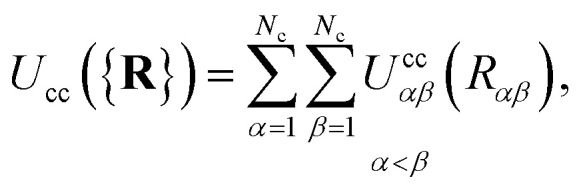
where *R*_*αβ*_ is the magnitude of the center-to-center separation vector between the *α*th and *β*th colloids (*i.e.*, *R*_*αβ*_ = |**R**_*α*_ − **R**_*β*_|). Furthermore,20
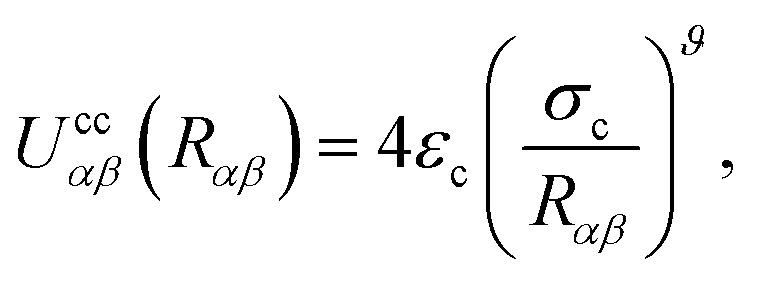
where *ε*_c_ represents the interaction strength between colloid particles, *σ*_c_ denotes the colloid diameter, and *ϑ* controls the softness of the repulsive interaction. For all colloid-pair samples, we used *ε*_c_ = *ε*_0_, *σ*_c_ = 7*σ*_e_, and *ϑ* = 36 (*i.e.*, *ε*_0_ = 1.0 and *σ*_e_ = 1.0, as indicated in Section 2.1).

As is well known, colloids can undergo clustering or form aggregates through short-range attractive forces (*e.g.*, van der Waals interactions), as well as through dipolar or quadrupolar attractive effects.^11^ Because our interest is in discerning the ability of the nematic LC in producing effective attractions between colloidal particles, it is important to use the form provided in eqn (20), which neglects any attractions due to colloid–colloid interactions. We thus ensure that any colloid–colloid attractive interactions are solely due to the underlying LC solvent.

## 3 Methods

Details on implementing the model in computer simulations and the formalisms for analyzing system properties are summarized in this section. As discussed in the opening remarks of Section 2, two types of systems were investigated in this work: single-colloid and colloid-pair samples. In both scenarios, colloidal inclusions were contained in a nematic-phase discotic solvent.

### 3.1 Molecular dynamics simulations

Trajectories for all samples were acquired with Molecular Dynamics (MD) simulations performed in the canonical (*NVT*) ensemble. To implement the colloid–LC model, eqn (1) was generalized to include a time variable *t*: *U*_tot_({**r**(*t*)},{**ê**(*t*)}, {**R**(*t*)}). Simulations were parallelized to expedite the time evolution of all samples. To further optimize the simulations, neighbor lists were used with distance cutoffs as follows: for the mesogen–mesogen contribution, *r*_cut_ = 1.6*σ*_e_; for the mesogen–colloid contribution, *X*_cut_ = *σ*_c_ + *σ*_f_; for the colloid–colloid contribution, *R*_cut_ = *σ*_c_ + *σ*_e_. Equations of motion were integrated with the velocity-Verlet algorithm, using a simulation time step of *δt* = 0.001 for translational and orientational dynamics.^100,101^ A typical simulation consisted of 
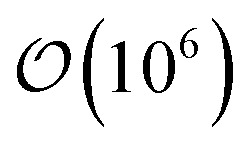
 time steps.

Samples consisted of 
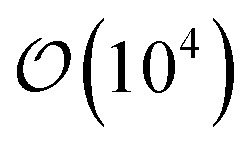
 discogens contained in 3D cubic or rectangular cells. Periodic boundary conditions were applied in all spatial dimensions. Thus, each system represented an unconfined sample of a discotic solvent with colloidal inclusions. For single-colloid samples, *N*_m_ = 28 000 discogens were contained in a simulation cell with dimensions *L*_*x*_ = *L*_*y*_ = *L*_*z*_ = 22*σ*_e_. For colloid-pair samples, *N*_m_ = 56 000 discogens were contained in a simulation cell with dimensions *L*_*x*_ = *L*_*y*_ = 22*σ*_e_, *L*_*z*_ = 44*σ*_e_. Colloidal dispersions were prepared with a number density *ρ* = 2.63 and temperature *T* = 7.0. For this state point, the observed pressure for the bulk solvent is *P* = 220, located deep in the nematic (*N*_D_) phase region for the Gay–Berne parameterization used in this study.^97,98^ The temperature for a given sample was held fixed using a Nosé–Hoover thermostat. A simulation run was performed independently for each value of *κ*′′ in the study (as described in Section 2.2).

The model presented in this work is in terms of reduced units. To avoid cumbersome notation, all reduced units have been denoted without an asterisk (contrary to convention).^102^ Thus, all dimensionalized quantities are reported in “star notation”, *e.g.*, t* refers to a time in ps. As a reference experimental system, we used triphenylene hexa(heptoxybenzoate)^103^ [abbreviated as C7OHBT^91^ or H7OBT^94,104^] for several reasons: (a) it contains the original mesogenic core (*i.e.*, triphenylene) of the Gay–Berne parameterization,^89,105^ (b) it exhibits the discotic nematic (*N*_D_) mesophase relevant in this work,^84,106^ and (c) its physically-relevant model parameters have been previously reported. Similar parameter sets have been used to expand the range of studies possible with computer simulations to model specific phenomena in discotic liquid crystals.^107^ The coarse-grained representation adopted in this work can be approximately dimensionalized using 
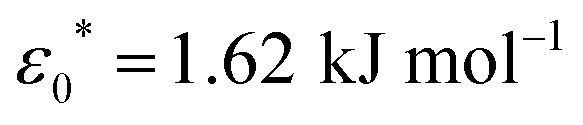
, 
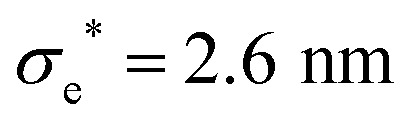
, and m* = 1634.064 g mol^−1^.^91^ With these units, key system variables are found as follows: t* = (82.6 ps)*t*, T* = (194.8 K)*T*, P* = (1.53 bar)*P*, and ρ* = (0.0569 nm^−3^)*ρ*. For example, the dimensionalized simulation time step is approximately equivalent to δt* = (82.6 ps)*δt* = (82.6 ps)(0.001) = 82.6 fs.

### 3.2 Orientational order

To characterize interactions that emerge between solvent and colloidal inclusions, the local orientational order of the LC solvent was determined from the Maier–Saupe (nematic) order parameter *S*_2,loc_, obtained by diagonalizing the orientational tensor,21
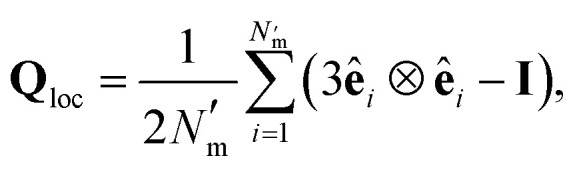
where ⊗ denotes the tensor product, **I** corresponds to the identity matrix, and 
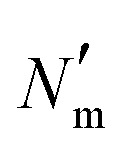
 is the number of mesogens contained in a subvolume of the ensemble [chosen to be sufficiently large such that 
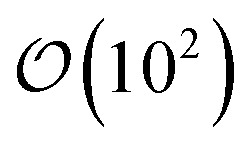
 discogens]. Because our analysis accounts for discogen subpopulations (*i.e.*, through discretized volumes), finite-size corrections are implemented as reported previously.^108–110^ For an isotropic sample, *S*_2,loc_ = 0. As the population of molecular principal axes (*i.e.*, normal to the plane of the discogen face) aligning with the director in the region **n̂**_loc_ increases, *S*_2,loc_ will also increase. The extension of eqn (21) to include all *N*_m_ discogens in the ensemble yields *S*_2_ = *λ*_max_ as the largest eigenvalue (after diagonalizing **Q**);^111^ the corresponding normalized eigenvector is the director **n̂** of the entire sample.

Local orientational order is presented in color maps conveying a transverse plane containing the center-to-center vector of the colloid pair and parallel to one of the faces of the rectangular simulation cell. The local nematic order parameter is averaged over 
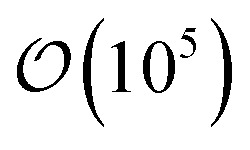
 equilibrium simulation steps. Color maps are derived by gridding data from the local orientational order to obtain subregions in the system, with a linear resolution of 0.33*σ*_e_.

### 3.3 Free energy

For colloid-pair systems, the potential of mean force (PMF) between the colloidal inclusions immersed in a nematic-phase discotic solvent is used to characterize the effective attraction between the dyad. It is stressed that such an attraction is prompted by the underlying solvent only, and not the result of any type of covalent bonding. To perform the calculation, the position vector of a reference colloid (*i.e.*, **R**_1_) is fixed while systematically changing the position vector of the probe colloid (*i.e.*, **R**_2_). The calculation is initiated with a sufficiently large separation *R*_12_ = |**R**_1_ − **R**_2_|, such that the force acting on the colloid pair corresponds to that of the bulk solvent (*i.e.*, remains constant). As a next step, *R*_12_ is decreased in *N*_s_ steps (*e.g.*, this amounts to *N*_s_ ∼ 26 steps in our study, from *R*_12_ ∼ 9.5*σ*_e_ to 7.0*σ*_e_). For each step, the force acting between colloidal inclusions is recorded multiple times from which the mean value is determined at the end of the run. The separation between colloids is constrained for a given value of *R*_12_ in order to acquire a statistically meaningful average. The calculation is complete when *R*_12_ ≈ *σ*_c_. Integrating the mean force over the trajectory *ξ* of *N*_s_ steps yields the PMF acting between the colloid pair.^112^

Because the distance *R*_12_ is the order parameter of the PMF, the mean force *F*(*R*_12_) acting between the colloid pair is22
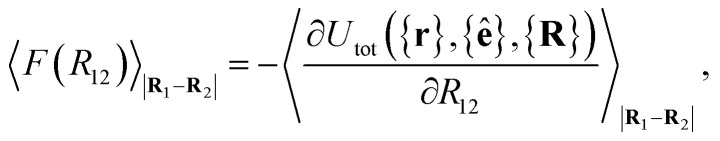
where 〈⋯〉_|**R**_1_−**R**_2_|_ signifies an average over all configurations in which the separation between **R**_1_ and **R**_2_ matches *R*_12_. In addition, the mean value 〈*F*(*R*_12_)〉_|**R**_1_−**R**_2_|_ is obtained by averaging over all discogen coordinates and all energy contributions involving the colloid pair. Therefore,23
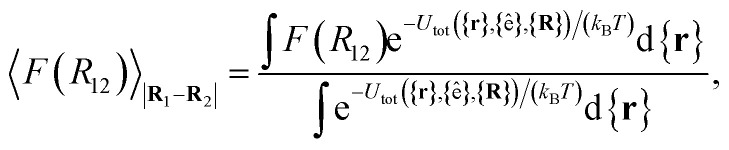
where *k*_B_ is the Boltzmann constant and the set of differentials d**r**_1_d**r**_2_…d**r**_*N*_m__ in the integral for the canonical average has been shortened to d{**r**}. Recognizing the denominator of eqn (23) as the configurational partition function *Q* and that the PMF is related to the Helmholtz free energy *A* as

24 *A*(*R*_12_) = −*k*_B_*T*  ln *Q*(*R*_12_),

it can be established that25
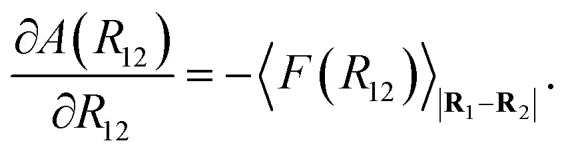
As the distance between colloids *R*_12_ is systematically changed (while collecting an average force over the trajectory *ξ*), each *i*th step |**R**_1_ − **R**_2_|_*i*_ of the *N*_s_ steps corresponds to a different system with a different free energy.^112^ The change in free energy between two steps (*e.g.*, *i* + 1 and *i*) is then given by

26 Δ*A*(*R*_12_)_*i*+1,*i*_ = *A*(*R*_12_)_*i*+1_ − *A*(*R*_12_)_*i*_.

The change in free energy (as a function of *R*_12_) is then27
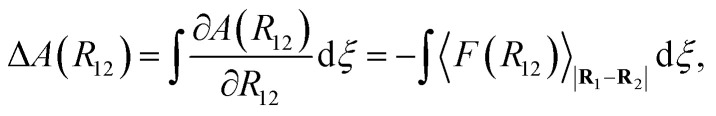
where d*ξ* represents a trajectory differential [*i.e.*, *ξ* is the path for the probe colloid (*i.e.*, *R*_2_) to approach the reference colloid (*i.e.*, *R*_1_), so *ξ* is a function of *R*_12_].

## 4 Results and discussion

Results are presented separately for single-colloid and colloid-pair systems. For colloid-pair systems, the intercolloid (center-to-center) vector **R**_12_ can take on an arbitrary orientation with respect to the nematic-field director **n̂**. Two limiting scenarios are considered here when initializing the system: when **R**_12_ is parallel to **n̂** (*i.e.*, the PARA case) and when **R**_12_ is perpendicular to **n̂** (*i.e.*, the PERP case). In both scenarios, **R**_12_ is arranged to be parallel to the *z*-axis (*i.e.*, the longest dimension of the rectangular simulation cell) when showing simulation snapshots, unless otherwise noted. In all cases, the orientation of the far-field director is conveyed in a sphere-referenced coordinate system. The resulting equilibrium arrangement for the two orientation scenarios are treated separately.

### 4.1 Single-colloid systems

**Fig. 3:**
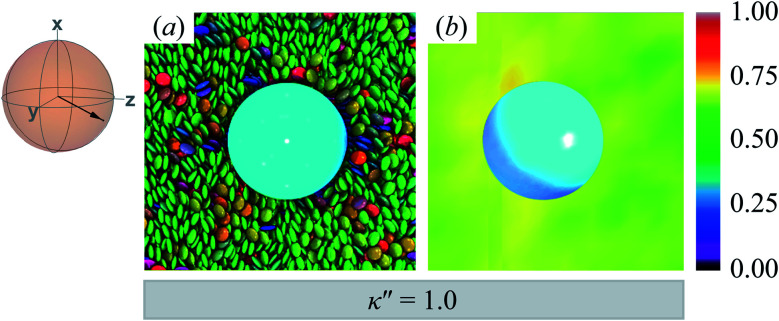
Equilibrium configuration for a single-colloid system with an anchoring strength anisotropy of *κ*′′ = 1.0. (a) Cross-section in the *yz*-plane with respect to the colloidal particle in the discotic solvent. Each discogen is colored according to its orientation vector. The average orientation of the far-field director for the system at equilibrium is indicated by the sphere-referenced coordinate system. (b) Color map of the local nematic order parameter *S*_2,loc_, in the same plane as in Panel (a): no topological defect is observed. The color bar is used to characterize *S*_2,loc_ within a local region on the color map. Refer to Section 3.2 for computational details of the color map. All colloidal dispersions in this study were prepared with fixed number density (*ρ* = 2.63) and temperature (*T* = 7.0), giving a bulk internal pressure of *P* = 220. Under these conditions, the Gay–Berne model used in this study is located deep in the nematic (*N*_D_) phase.^97,98^

The reference system for single-colloid samples is characterized by discogens that have no preference for either homeotropic or planar anchoring (*i.e.*, *κ*′′ = 1). Results for the reference system are shown in Fig. 3(a), where no prevalent color is observed for discogens in contact with the colloid surface. Bulk regions of the solvent mostly show the same (green) color, indicating a favored orientation characteristic of the *N*_D_ phase. The corresponding orientational order color map in Fig. 3(b) shows that the nematic order parameter fluctuates between *S*_2,loc_ = 0.50–0.75, typical of the *N*_D_ phase, with no perceptible defect.

**Fig. 4:**
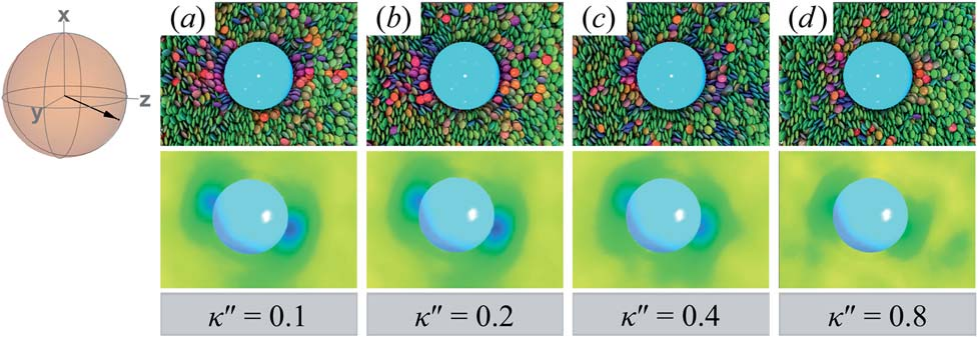
Equilibrium configurations in the *yz*-plane for an energy anisotropy favoring parallel anchoring: *κ*′′ = {0.10, 0.20, 0.40, 0.80}. Top panels are as in Fig. 3(a), whereas bottom panels are as in Fig. 3(b). Two opposite regions appear on the colloid surface with orientational disorder (*i.e.*, blue and green shading in the color maps). The effect diminishes as *κ*′′ → 1. The average orientation of the director in the sphere-referenced coordinate system applies to all samples.

Topological defects become pronounced as planar anchoring anisotropy increases (*i.e.*, *κ*′′ → 0). This can be appreciated in sample cross-sections of Fig. 4 (top panels), comparing data for *κ*′′ = 0.8 to 0.1. Discogen colors near the colloid surface exhibit planar anchoring (*i.e.*, when discogen edges interact with the colloid surface). When *κ*′′ = 0.1, the planar anchoring energy overwhelms the competing potential energy of the nematic field: discogens align perpendicular to the director at the equatorial poles of the colloid, giving rise to the formation of boojums. The defect is easily traced in the order parameter color maps of Fig. 4 (bottom panels). The nematic order parameter (*S*_2,loc_ ∼ 0.3) is lower for boojums when compared to that of the *N*_D_ phase.

**Fig. 5:**
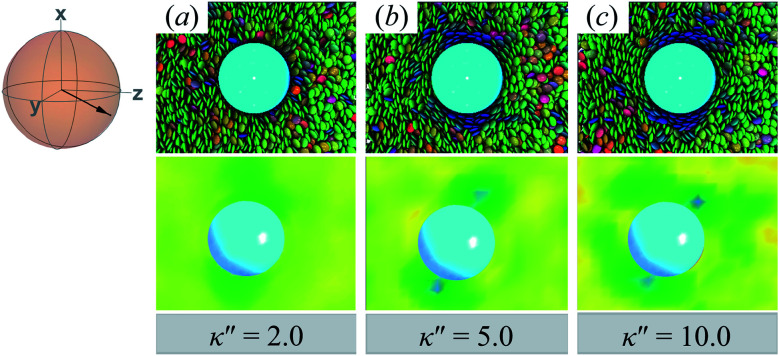
Same as in Fig. 4, but for an energy anisotropy favoring homeotropic anchoring: *κ*′′ = {2.0, 5.0, 10.0}. Although the color maps share some similarities to those in Fig. 4, they in fact correspond to Saturn ring defects.

Results for homeotropic anchoring (when *κ*′′ > 1) are shown in Fig. 5. As expected, sample cross-sections show that discogen faces osculate the colloid surface, an effect that becomes more pronounced as *κ*′′ increases from unity. The effect of anchoring can be gauged by the color change in discogens near the colloid surface, shown in the top panels of Fig. 5: purple discogen hues are pronounced at the meridian poles of the colloid: the discogens reorient perpendicularly relative to the nematic-field director. Color maps of the nematic order parameter are shown in the bottom panels of Fig. 5. In the bulk regions of the solvent, the orientational order parameter fluctuates around values characteristic of the *N*_D_ phase (*i.e.*, *S*_2,loc_ ∼ 0.50–0.75). As homeotropic anchoring becomes more favorable, regions with low values of the nematic order parameter (*i.e.*, *S*_2,loc_ ∼ 0.25) also increase, indicating the onset of orientational frustration driven by topological defects.

**Fig. 6:**
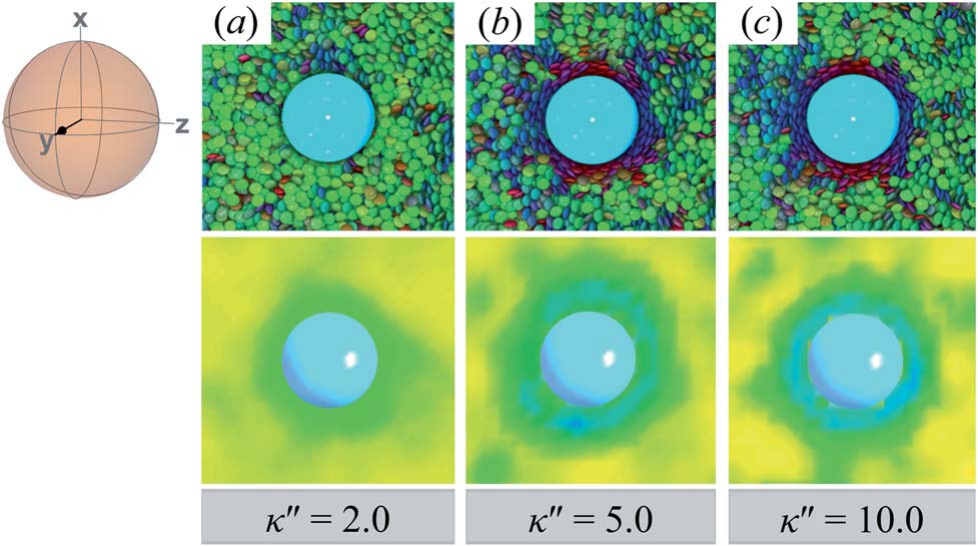
Same as in Fig. 5, but for the *xy*-plane. Saturn rings are evident in this cross-sectional view.

Topological defects from the color maps in Fig. 5 appear to be similar to those in Fig. 4, but are in fact due to a different topological defect: a Saturn ring. Thus, the concentric-like blue shading observed with boojums is lost. An alternate cross-sectional view of the sample is presented in Fig. 6, where the ring structure becomes apparent. Colloidal inclusions immersed in nematic-phase prolate systems (*i.e.*, *σ*_e_ < *σ*_f_) for which homeotropic anchoring is favored also display Saturn rings.^79,113^ In our systems, topological defects become stable against thermal fluctuations when *κ*′′ > 2. When 1 ≤ *κ*′′ ≤ 2, defects are not well defined, as can be inferred from Fig. 6.

**Fig. 7:**
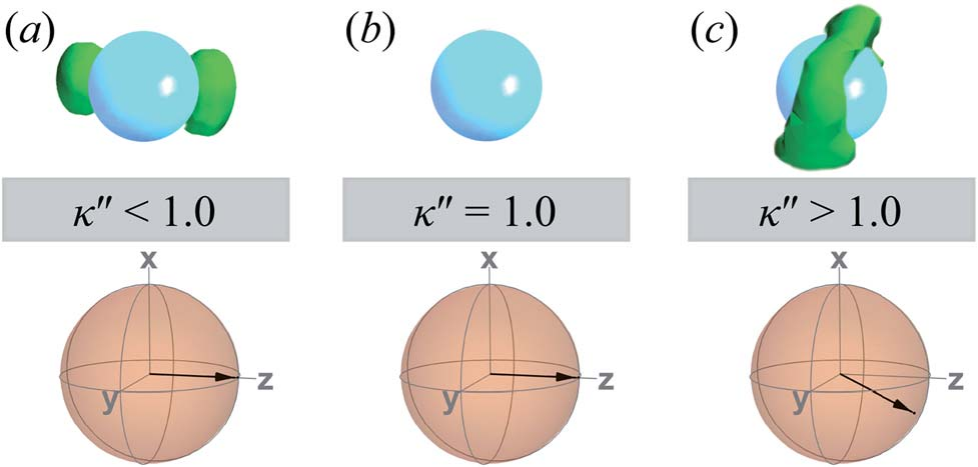
A visual summary of topological defects for single-colloid systems in a *N*_D_ solvent: (a) boojums form with planar anchoring [*i.e.*, *κ*′′ < 1], (b) no discernible defects arise when no anchoring type is energetically preferred [*i.e.*, *κ*′′ = 1], and (c) Saturn rings emerge with homeotropic anchoring [*i.e.*, *κ*′′ > 1]. The average orientation of the director is shown below each rendition, indicated by the sphere-referenced coordinate system.

A visual summary on the topological defects observed in single-colloid systems is provided in Fig. 7. The left panel (*i.e.*, *κ*′′ < 1) shows that boojums appear when planar anchoring is favored (*i.e.*, *κ*′′ < 1), which manifest on the colloid surface on opposite ends [*cf.*Fig. 7(a)]. When there is no preference in anchoring mode (*i.e.*, *κ*′′ = 1), no topological defects appear in the system [*cf.*Fig. 7(b)]. Lastly, Saturn rings emerge when homeotropic anchoring is favored (*i.e.*, *κ*′′ > 1) [*cf.*Fig. 7(c)].

### 4.2 Colloid-pair systems

In this section, colloid-pair samples are treated. All simulation snapshots show an average final (surface-to-surface) distance between colloids of 1.7(±0.2)*σ*_e_. The PARA and PERP cases are treated separately in the following subsections; refer to the opening remarks of Section 4 for a definition of these two cases.

Colloidal dyads do not possess a bond of any sort in this work: each colloid is free to undergo independent translational motion, subject to the excluded volume of other colloidal particles and the discotic solvent. Additionally, a colloidal dyad is not constrained or confined to attain an equilibrium arrangement. Furthermore, there are no external fields used to constrain the relative orientation between the intercolloidal vector and the far-field director attained at equilibrium.

Although the PARA and PERP designations strictly describe initial configurations of different systems, they are still useful when classifying spatial arrangements observed at equilibrium due to the distinctly identifiable behavior in each case. Although the relative orientation between the intercolloidal separation vector and the far-field director (*i.e.*, angle) is conserved, their absolute position in the simulation cell is subject to change. All simulation snapshots shown in this discussion were centered and adjusted so that the intercolloidal vector is parallel to the *z*-axis of the simulation cell in order to ease comparison between different anchoring modes and arrangements.

#### 4.2.1 Parallel configuration

**Fig. 8:**
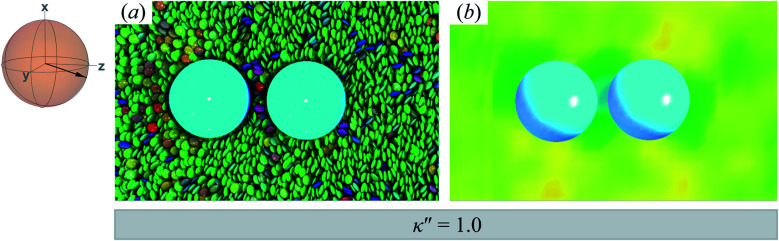
As in Fig. 3, but for a colloid-pair immersed in a *N*_D_ solvent. The director **n̂** is initialized to be parallel to the intercolloid vector **R**_12_ (*i.e.*, PARA case). When there is no anchoring preference in the system (*i.e.*, *κ*′′ = 1), the nematic field stabilizes the colloid dyad through periodic, fleeting coupling of boojum defects.

As was done for single-colloid systems, the reference system in which there is no preference in anchoring (*i.e.*, *κ*′′ = 1) is considered first. On inspecting the arrangement of discogens throughout the colloid-pair system in Fig. 8(a), there appears to be no preferential ordering on the colloidal surfaces: the solvent occupies the intercolloid region with no preferred orientation. However, the color map of the corresponding configuration, Fig. 8(b), shows that weak but evident defects are present. More specifically, two boojum defects (one set on each colloid) emerge slanted, following the direction of the nematic-field director. Within the intercolloid region, the boojum from one colloid coalesces with that of the other colloid. This behavior is only fleetingly stable when *κ*′′ = 1, as inferred from a visual inspection of the associated MD trajectory. As for the bulk regions of the system, the color map establishes that discogens, on average, orient along the nematic-field director. The local-field director was assessed for two cases in the limit of strong anchoring: bulk-like homogeneity is recovered beyond the spatial domain of the colloidal-pair inclusions. Sample calculations are deposited as ESI† for this work (see ESI† file Local-Field.pdf).

**Fig. 9:**
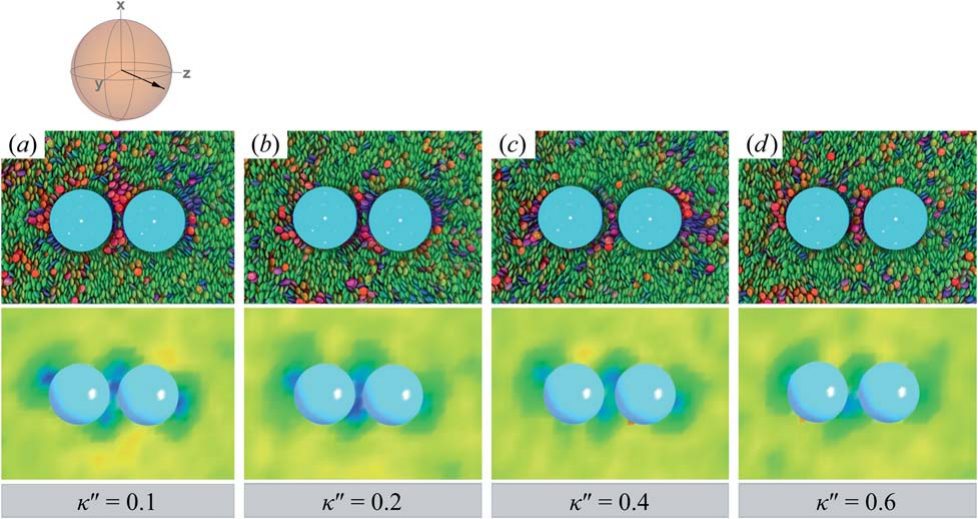
As in Fig. 8, but for *κ*′′ = {0.1, 0.2, 0.4, 0.6} (P-PARA case). The coupling of boojum defects becomes more stable against thermal fluctuations as the anchoring strength anisotropy increasingly favors planar anchoring (*i.e.*, as *κ*′′ decreases). The average orientation of the director in the sphere-referenced coordinate system applies to all samples.

As planar anchoring becomes more favorable, upon decreasing *κ*′′ from unity, the colloid surface energy overcomes the field imposed by the nematic phase and the formation of boojums is now stabilized when compared to samples with *κ*′′ = 1, as was the case for single-colloid systems. We term this arrangement the P-PARA case. An important difference with the colloidal dyad is that the system minimizes the internal energy by having colloids “share” common, orientationally-disordered zones. Configuration snapshots and order parameter color maps for this scenario are shown in Fig. 9. Effectively, the “sharing” of defects leads to attractive interactions between colloidal inclusions in which colloidal assembly is mediated by topological defects.

**Fig. 10:**
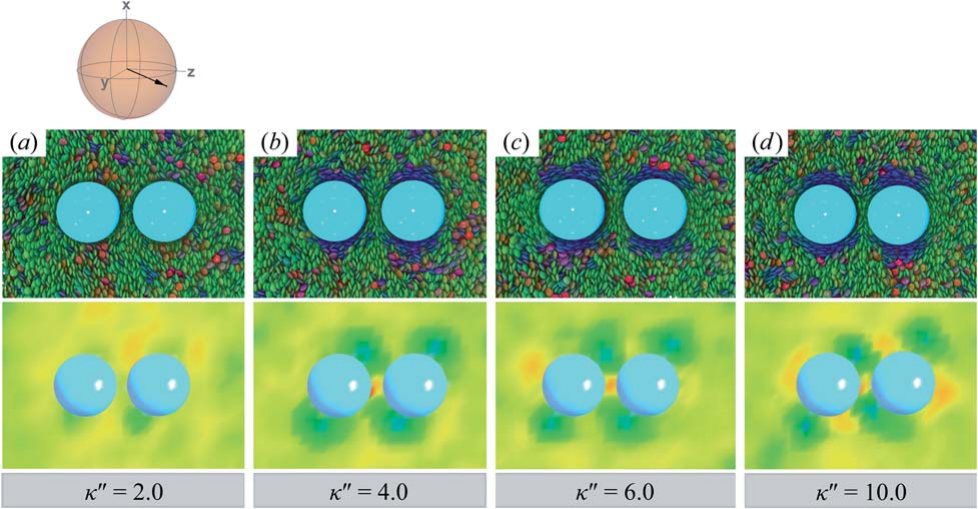
As in Fig. 9, but for *κ*′′ = {2.0, 4.0, 6.0, 10.0} (H-PARA case). Although these snapshots are reminiscent of the coupled boojums in Fig. 8, the defects actually correspond to a pair of slanted Saturn rings. This arrangement is better discerned in Fig. 11.

When increasing *κ*′′ from unity to induce homeotropic anchoring, an arrangement denoted here as the H-PARA case, Saturn rings appear in a manner consistent with the single-colloid samples. Configuration snapshots and order parameter color maps for this regime are shown in Fig. 10. In similar fashion to the P-PARA case, the Saturn rings in the H-PARA case appear with a slanted geometry, following the orientation of nematic-field director (*i.e.*, a two o'clock slant, *versus* the ten o'clock slant of the P-PARA case). The difference in how the systems were initialized leads to qualitatively different interactions between defects. In the P-PARA case, defects merge in a sustained manner with time; in the H-PARA case, Saturn rings coalesce in subregions sporadically and transiently.

**Fig. 11:**
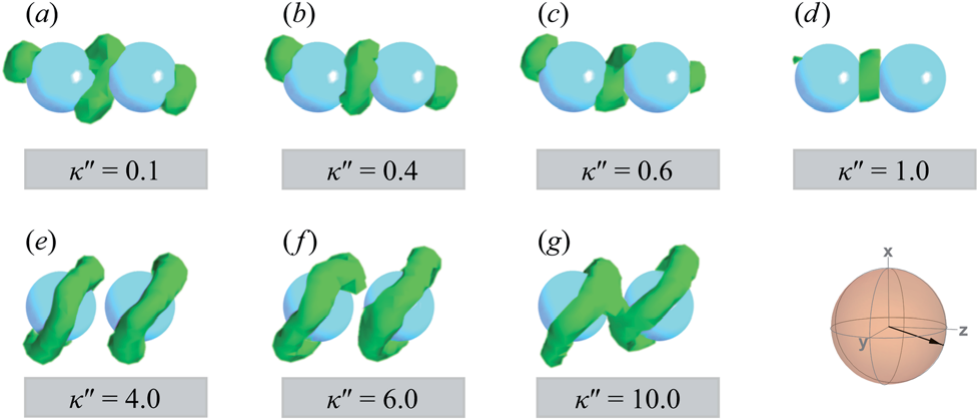
A visual summary of topological defects for colloid-pair systems initialized with a PARA arrangement, with anchoring anisotropies *κ*′′ as indicated. Planar anchoring [Panels (a), (b), and (c)] leads to a of coupling boojums. The boojums on each colloid engage even when no specific anchoring is preferred [Panel (d)]. Homeotropic anchoring [Panels (e), (f), and (g)] leads to Saturn ring pairs. The average orientation of the director in the sphere-referenced coordinate system applies to all samples.

The slanted arrangement of topological defects, as observed here for the P-PARA or H-PARA cases, has been observed previously in the literature for calamitic systems.^15,64,83,114–119^ Both Saturn rings and boojums are quadrupolar defects^120^ and thus engage with other particles *via* quadrupolar interactions, obeying characteristic conservation laws.^12,121^ This governs the manner in which dimers and higher-order structures form: zigzag or chain-like aggregates are stabilized by exhibiting a slight inclination with respect to the intercolloid vector. In essence, this reduces the volume of the distorted region with respect to the director^83^ and minimizes the overall energy of the system. A visual summary of the topological defects observed for colloid-pair systems (both P-PARA and H-PARA scenarios) is provided in Fig. 11.

To qualitatively assess the temporal behavior of topological defects, we inspected trajectories borne from the MD simulations. Trajectories were visualized and deposited as ESI† for this work. In the P-PARA case, the intercolloid domain is always populated with the topological defect. The intensity with which the defect across the intercolloid domain is shared varies with time. At the periphery of the colloidal dyad, defects are significantly more mobile and their intensity is just as variable (see ESI† file P-PARA.mp4). For the H-PARA case, the shape and slant of the Saturn rings are persistent. With time, the rings fuse transiently along a subregion within the intercolloid domain: this flickering behavior, however, does not yield complete “fusion” of the Saturn ring dyad (see ESI† file H-PARA.mp4).

#### 4.2.2 Perpendicular configuration

**Fig. 12:**
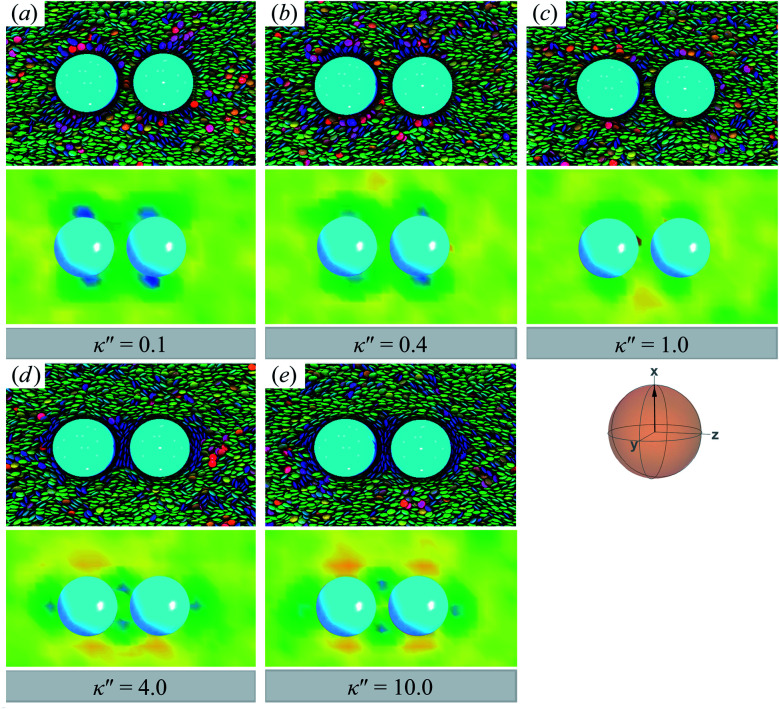
As in Fig. 9 and 10, but when the director **n̂** is initialized to be perpendicular to the intercolloid vector **R**_12_ (PERP case). Planar anchoring [Panels (a) and (b)] leads to a parallel arrangement of boojums (the P-PERP case), which align with the nematic-field director. Even when neither type of anchoring is favored [Panel (c)], the PARA arrangement of boojums is operative. Homeotropic anchoring [Panels (d) and (e)] leads to a triad of Saturn rings: two coplanar rings perpendicular to **n̂** adjoined by a third ring perpendicular to both the intercolloid vector **R**_12_ and to the plane containing the other two rings. This H-PERP case is better discerned in Fig. 13.

**Fig. 13:**
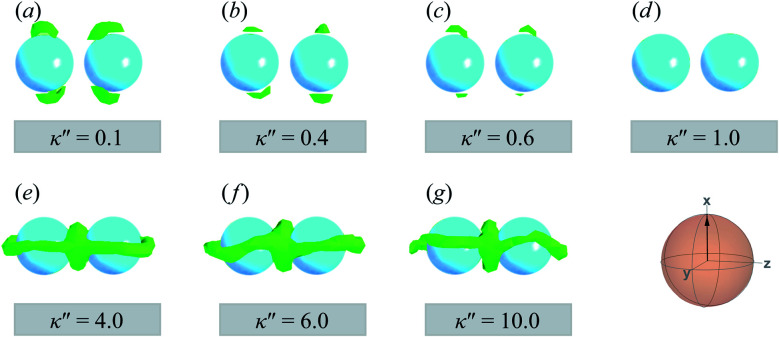
As in Fig. 11, but for the PERP case. The trends correspond to those already described in Fig. 12. The Saturn ring triad described in Fig. 12 is more evident in Panels (e), (f), and (g). For *κ*′′ = 1, the formation of boojums is imperceptible in Panel (d), but evident in Panel (c) of Fig. 12: this hints at the highly dynamic nature of the defect.

In this section, focus is given to the PERP case for colloid-pair samples. Results for both planar and homeotropic anchoring appear in Fig. 12. As expected, the color maps show the appearance of boojums for planar anchoring (*i.e.*, *κ*′′ = 0.1, 0.4). Specifically, the boojums are mutually parallel and align with the nematic-field director in the P-PERP case. However, the extent of interaction between the set of boojums is sporadic and it is not sustained within the intercolloid gap, as in the P-PARA case. When there is no preference in anchoring (*i.e.*, *κ*′′ = 1), the system already presents a weak effect that is driven solely by the field of the nematic solvent [*cf.*Fig. 12(c), as was already seen for the P-PARA case]. For homeotropic anchoring (*i.e.*, *κ*′′ = 4.0, 10.0), a Saturn ring triad is observed: two coplanar rings perpendicular to **n̂** are adjoined by a third ring perpendicular to **R**_12_ and to the plane containing the other two rings. The result for the H-PERP case has been previously observed in prolate nematic colloids^15,79^ as well as in numerical studies^17,122^ and simulations.^113^ Shown in Fig. 13 is a visual summary of topological defects for the PERP case for the two anchoring energy anisotropies (*i.e.*, P-PERP and H-PERP).

Similar to the treatment of the dynamical assessment of defects in Section 4.2.1, the P-PERP and H-PERP cases are pursued here. For planar anchoring, the parallel boojum arrangement presents a flickering behavior, but seemingly the intercolloid domain never mediates the topological defect (see ESI† file P-PERP.mp4). Boojums “fuse” transiently from the top or bottom region, in a sideways manner. On the other hand, the Saturn ring triad observed for homeotropic anchoring is stable throughout, showing characteristic solvent fluctuations (see ESI† file H-PERP.mp4). This latter behavior is of interest given that theoretical^122^ and simulation studies with prolate mesogens^17^ indicate that the stability of the Saturn ring triad may be metastable or induced by confinement, an effect sensitive to the relative length scales of the system. Although finite-size effects may be operative in these systems, they are still experimentally relevant given that many LC-based applications have spatial design constraints. Additionally, numerical studies have found that the Saturn ring triad is relatively short-lived:^17^ we found no indication that this structure is transient on the timescales considered here, nor that it is a time-averaged artifact^113^ as shown by the ESI† files.

The dynamical behavior of topological defects, as previously discussed, provide insight into the elastic interactions prompted by the *N*_D_ solvent. For instance, fluctuations leading to the transient coalescence of defects across colloidal inclusions is a clear manifestation of the reversible, non-covalent attractive interactions cited as being requisite for molecular self-assembly.^4,5,123^ Colloidal structures mediated by LC solvents are thus driven by the system so as to minimize energy penalties. The reversible nature of these interactions is central to self-healing materials,^3^ in which structural aberrations can dissipate *via* a fluctuating background imposed by topological defects.

### 4.3 Free energy of colloid pair inclusions

**Fig. 14:**
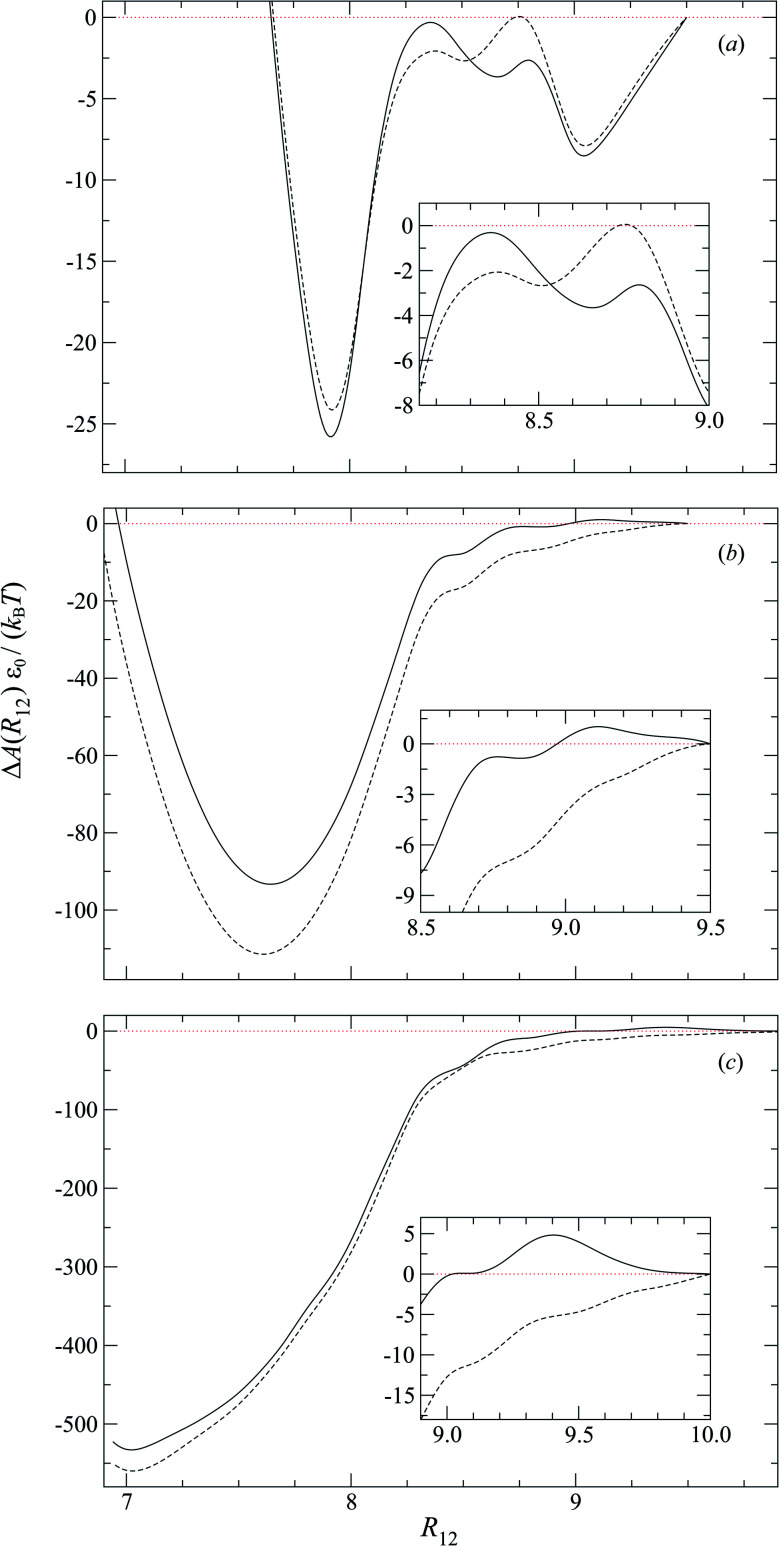
The normalized change in free energy Δ*A*(*R*_12_)*ε*_0_/(*k*_B_*T*) as a function of the intercolloid (center-to-center) separation *R*_12_ for different values of anchoring strength anisotropy: (a) *κ*′′ = 0.10, (b) *κ*′′ = 4.0, and (c) *κ*′′ = 10.0. Shown are PARA (dashed line) and PERP (solid line) cases. As a guide to the eye, the limit Δ*A*(*R*_12_) = 0 is shown (dotted red line). Insets are zoomed regions of Δ*A*(*R*_12_)*ε*_0_/(*k*_B_*T*) at larger intercolloid distances.

To characterize the effective interactions present in the colloid-pair samples, the change in free energy Δ*A*(*R*_12_) was determined as a function of intercolloid (center-to-center) separation *R*_12_. We remind the reader that the separation between colloids is constrained for a given value of *R*_12_ in order to acquire statistically meaningful averages for Δ*A*(*R*_12_). Focus is given to comparing the two limiting geometrical scenarios: the PARA and PERP cases. Results from these calculations are shown in Fig. 14.

**Fig. 15:**
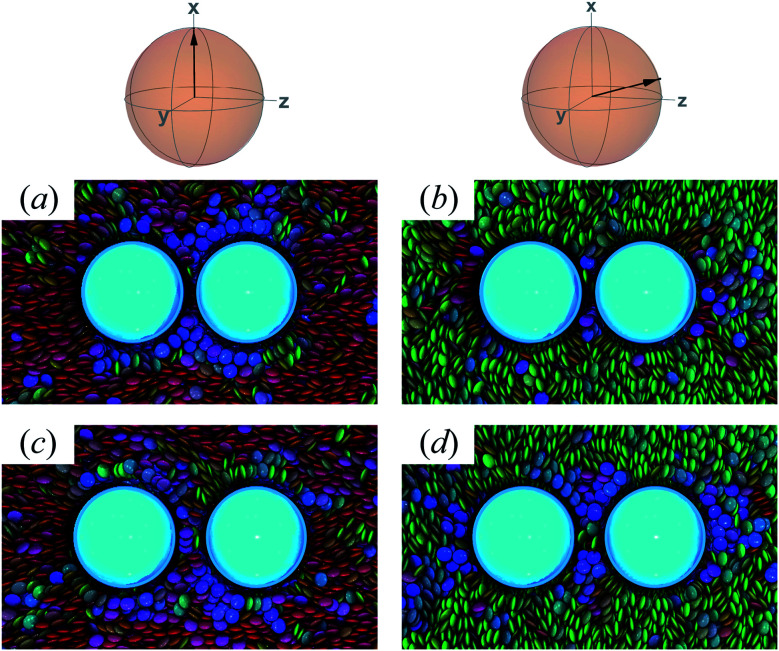
Configuration snapshots linked to global minima in the free energy profiles of Fig. 14(a) [*i.e.*, when *κ*′′ = 0.1]. To ease the distinction in the alignment of the director with respect to the intercolloid vector, the color scale for the discogens was adjusted: red samples correspond to the PERP case, whereas green samples are for the PARA case. Panels (a) and (b) are for *R*_12_ ∼ *σ*_e_; Panels (c) and (d) are for *R*_12_ ∼ 9*σ*_e_. The average orientation of the director in the sphere-referenced coordinate system applies to the two samples in each column.

When the colloid dyad engages through planar anchoring (*i.e.*, *κ*′′ = 0.1), the interaction is slightly more stable [*i.e.*, lower Δ*A*(*R*_12_)] for the PERP case. This can be gauged from the global minimum at *R*_12_ ∼ 8*σ*_e_ in Fig. 14(a). This length scale corresponds to a surface-to-surface gap between colloids of ∼*σ*_e_, which nominally would accommodate up to a discogen with its edges making contact between the two colloid surfaces. The gap is actually less due to an effective “halo” surrounding the colloid, produced by the mathematical form of the mesogen–colloid interaction (a shell of thickness ∼ 0.3*σ*_e_). Physically, the “halo” is an additional excluded volume allowing only for facial interaction (*i.e.*, the discogen “face” bridges the two colloidal surfaces within the gap), which dominates the global minimum in Δ*A*(*R*_12_). The magnitude of Δ*A*(*R*_12_) for this case is comparable to previously published studies on calamitic samples with colloidal inclusions.^78^ A similar response is observed for the PARA case, albeit with a slightly less stable [*i.e.*, higher Δ*A*(*R*_12_)] global minimum. Sample snapshots for both geometrical scenarios are provided in Fig. 15(a) and (b).

An additional local minimum is observed (with planar mesogen anchoring) when the intercolloid separation is *R*_12_ ∼ 9*σ*_e_: this corresponds to a surface-to-surface gap between colloids of ∼2*σ*_e_. After accounting for the colloidal “halos”, the gap is sufficiently wide to accommodate a discogen positioned so that its edges bridge the gap. Sample snapshots for both scenarios (*i.e.*, PARA and PERP) are shown in Fig. 15(c) and (d). Similar to the response observed for the global minimum, the local minimum is slightly more stable in the PERP case.

**Fig. 16:**
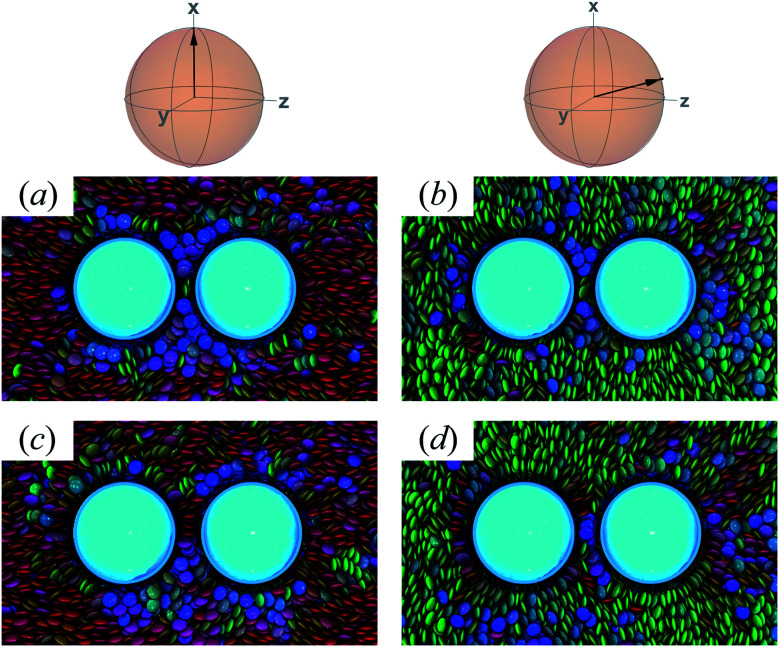
As in Fig. 15, but for the crossover region when 8.2*σ*_e_ < *R*_12_ < 8.9*σ*_e_. Panels (a) and (b) are for *R*_12_ ∼ 8.4*σ*_e_; Panels (c) and (d) are for *R*_12_ ∼ 8.8*σ*_e_.

The response in Δ*A*(*R*_12_) for 8.2*σ*_e_ < *R*_12_ < 8.9*σ*_e_ arises from the competition between energetically favorable mesogen–colloid interactions and the extent to which orientational order is transmitted from the bulk region of the solvent. In the lower end of the *R*_12_ range, there is a slightly higher energy penalty for the PERP case because the discogens in the intercolloid gap are distorted more sharply. This effect reverses in the higher end of the *R*_12_ range, where discogens within the gap “blend” their molecular directors with that of the bulk, with a concomitant lowering of Δ*A*(*R*_12_). These scenarios are contrasted in both limiting geometrical scenarios for *R*_12_ ∼ 8.4*σ*_e_ [Fig. 16(a) and (b)] as well as *R*_12_ ∼ 8.8*σ*_e_ [Fig. 16(c) and (d)].

For homeotropic anchoring (*i.e.*, *κ*′′ = 4.0 and *κ*′′ = 10), the only prominent feature is a global minimum for both PARA and PERP cases, at a length scale that tends toward intercolloid contact (*i.e.*, *R*_12_ ∼ 7*σ*_e_) with increasing *κ*′′. For the two values of *κ*′′ considered in this analysis, Δ*A*(*R*_12_) shows enhanced stability in the PARA case. This effect can be traced back to the manner in which Saturn rings are manifested in the two limiting geometries: in the PARA case, two slanted Saturn rings form, wherein transiently a subregion of the rings merges within the intercolloid gap. On the other hand, a Saturn ring triad arises in the PERP case: each colloid is surrounded by its own ring (coplanarly positioned, but perpendicular to the nematic-field director); the two resulting rings are joined by a third one positioned perpendicularly between them. These two scenarios are easily contrasted by revisiting Fig. 11(g) and 13(g). Although the ring-pair structure leads to greater stability [*cf.* the global minima in Fig. 14(b) and (c)], it has been difficult to compare this structure in the present literature: previous studies have focused on the PERP arrangement. The phenomenology observed in our study for the PERP case is consistent with previous work on calamitic systems.^78^

The study of free energy profiles proves useful in summarizing principles where the anchoring mode becomes a design control. Specifically, if colloidal inclusions are to assemble *via* the transient coupling of boojums, then an arrangement consistent with PERP geometry enhances the stability of such systems. On the other hand, if the coupling should occur through Saturn rings, the free energy analysis suggests that a PARA arrangement is relatively more stable, even though “communication” between rings is transient. At the expense of some stability, it is possible to attain an adjoined arrangement of Saturn rings with PERP geometry. These comments should be tempered against kinetic studies: it is possible that one arrangement might yield higher-order structures that are more readily attained dynamically. This issue is, however, beyond the scope of the present work.

## 5 Conclusions

Colloidal inclusions dispersed in a nematic-phase discotic (*i.e.*, oblate mesogen) solvent were studied at the level of Gay–Berne mesogens with Molecular Dynamics simulations. Gay–Berne parameters were tailored to capture a triphenylene core in a coarse-grained manner. Colloids were modeled as soft spheres with a purely repulsive interaction potential. Colloidal particles were coupled to the mesogenic solvent using a generalized interaction potential for mixtures of spherical and nonspherical particles. Systems were investigated containing one or two colloidal inclusions. Equilibrated trajectories from computer simulations were used to characterize the mesomorphic structure in the presence of the colloidal inclusions. Energy strengths in the colloid–mesogen description were adjusted to capture three anchoring modes: planar, homeotropic, and equally favorable.

In all cases, equilibrium configurations were acquired for unconfined systems in the absence of any external fields. The translational motion of colloidal particles was unconstrained when acquiring equilibrium data for solvent-mediated interactions. In the case of colloid-pair samples, each colloidal particle underwent independent motion. Furthermore, the relative arrangement between the intercolloidal separation vector and the far-field director is conserved but subject to typical thermal fluctuations.

Systems possessing a single colloidal inclusion yield either of two topological defects, depending on the anchoring mode. Planar anchoring leads to boojum defects, whereas homeotropic anchoring yields Saturn rings. No discernible topological defect is observed when either of these anchoring modes are equally favored from an energy standpoint, as expected. This is consistent with prior studies focused on calamitic (*i.e.*, prolate mesogen) liquid-crystalline solvents with colloidal inclusions.

A relevant issue when two colloidal inclusions are introduced into the nematic-phase discotic solvent is the effective interaction acting between the colloidal particles, which can lead to self-organization in the host solvent. To characterize the influence of the discotic solvent with respect to the far-field director, two limiting configurations were considered when initializing the system: the intercolloid separation vector of the colloidal pair is either parallel or perpendicular to the director. The pair of colloidal inclusions, when in close proximity, already exhibit boojums even when the colloid–mesogen interaction strengths do not favor one anchoring mode over another. This effect is solely driven by the nematic field of the solvent and is not observed when only one colloidal inclusion is present.

When adjusting the colloid–mesogen interaction strengths to favor either anchoring mode, the effective interaction between colloids will differ depending on the relative alignment of the director. Thus, four scenarios emerge: planar-parallel (P-PARA), planar-perpendicular (P-PERP), homeotropic-parallel (H-PARA), and homeotropic-perpendicular (H-PERP). Boojums are always observed to be axially parallel to the far-field director. Saturn rings appear with homeotropic anchoring and couple in a manner dependent on the relative arrangement of the colloid pair. Saturn rings arrange in parallel planes perpendicular to the far-field director. A triad of Saturn rings is observed in the H-PERP case: two coplanar rings perpendicular to the director are adjoined by a third ring that forms in the intercolloid domain, but parallel to the director.

The manner in which the nematic-phase discotic solvent drives an effective interaction between colloidal particles was assessed with free energy profiles. The order parameter for this calculation is the intercolloidal distance: the relative distance between a dyad is fixed for each value in the sweep of the order parameter to obtain statistically sound averages. When boojum defects arise in the system, colloids interact more favorably in the P-PERP case. When Saturn rings are involved, the interaction between colloids is more favorable in the H-PARA case. This orientation has been difficult to trace in the literature since most studies have previously focused on perpendicular arrangements.

Configuration snapshots and free energy profiles used as aids to characterize mesomorphic behavior are static in nature. Equally important is having access to dynamical information of such systems. To glean qualitative dynamical information of the topological defects due to colloidal-pair inclusions, trajectories from Molecular Dynamics simulations were inspected. Temporal windows were visualized from equilibrated samples for the four scenarios previously discussed. With the exception of the Saturn ring triad observed in the H-PERP case, defects display high spatial and temporal variation, and thus appear to “flicker”. In the P-PARA case, boojums merge in the intercolloid region. In the P-PERP case, the intercolloid region is not populated; instead, boojums merge periodically, for brief episodes, in a side-to-side fashion. The Saturn ring dyad in the H-PARA case, in which each colloid possesses its own ring, merges over a small region within the intercolloidal gap, briefly and periodically. The Saturn ring triad appearing in the H-PERP case yields a structure that persists spatially, with only thermal fluctuations being evident.

The ability to control topological defects is a relevant design parameter in many liquid-crystalline-based technologies. As our results show, the extent to which topological defects engage is a sensitive function of both anchoring mode and the relative arrangement of the nematic-field director with respect to colloidal inclusions. This work has combined static and dynamic information to better establish how effective interactions prompted by the nematic solvent aid in structuring colloidal inclusions. Our focus has been on a discotic solvent, given that it has not been previously used in the context of the issues we pursued here. The versatility of discotic systems, however, can extend the range of application given their unique mesophases and wider working range. It is anticipated that new modes to colloidal association may be possible in discotic solvents, in part because the molecular aspect ratio imparts a phase morphology different from that of calamitic systems. Future work in our group will focus on extending the complexity of colloidal assembly and quantitative dynamical analysis attainable from computer simulations.

## 6 Description of the ESI

The orientation of the local-field director throughout a sample was explored in the limit of strong anchoring for the PARA geometry. Results are reported in ESI† file Local-Field.pdf. Other anchoring strengths as well as the PERP cases explored in this work yield comparable results. The nematic field recovers bulk-like homogeneity beyond the spatial domain of colloidal inclusions.

Trajectories from equilibrated samples were visualized over a sufficiently long time interval to capture characteristic fluctuations of topological defects in nematic LC–colloid systems, for four characteristic scenarios. In each scenario, only the topological defect and the colloidal inclusions are shown for clarity; views were chosen to highlight the manner in which defects engage with a colloidal pair. Each video is differentiated by its anchoring mode and relative orientation (*i.e.*, between the nematic field director **n̂** and the intercolloidal separation vector **R**_12_):

• H-PARA.mp4.

Homeotropic anchoring (*κ*′′ = 10.0), when **n̂** is initialized to be parallel to **R**_12_.

• H-PERP.mp4.

Homeotropic anchoring (*κ*′′ = 10.0), when **n̂** is initialized to be perpendicular to **R**_12_.

• P-PARA.mp4.

Planar anchoring (*κ*′′ = 0.10), when **n̂** is initialized to be parallel to **R**_12_.

• P-PERP.mp4.

Planar anchoring (*κ*′′ = 0.10), when **n̂** is initialized to be perpendicular to **R**_12_.

Although colloidal inclusions do not appear to undergo significant displacements in the videos, each colloid experiences typical thermal fluctuations imposed by the underlying discotic solvent. Translational motion of each colloid is entirely unconstrained (*i.e.*, there are no covalent bonds between colloids that would keep their relative distance and/or orientation with respect to the far-field director constant).

## Conflicts of interest

There are no conflicts to declare.

## Supplementary Material

RA-009-C9RA05377H-s001

RA-009-C9RA05377H-s002

RA-009-C9RA05377H-s003

RA-009-C9RA05377H-s004

RA-009-C9RA05377H-s005
