# Supplementary material for: Defect-mediated colloidal interactions in a nematic-phase discotic solvent
Source: RSC Adv. 2019 Oct 17;9(57):33413–27. doi: 10.1039/c9ra05377h (PMC9073280; doi:10.1039/c9ra05377h)
Supplement: RA-009-C9RA05377H-s003 [file RA-009-C9RA05377H-s003.pdf]

Electronic Supplementary Material (ESI) for RSC Advances.
This journal is © The Royal Society of Chemistry 2019

Calculation of the Local-Field Director

Commensurate with the length scale used to generate order parameter color maps (refer to **Section 3.2** of the article for a description), the local-field director was explored for two limiting anchoring cases with the PARA geometry. The goal behind this calculation was to ensure that the nematic field presents uniform behavior far from colloidal inclusions and thus, the system recovers bulk-like behavior. Results are shown in Fig. S1 below.

(*a*) (*b*)

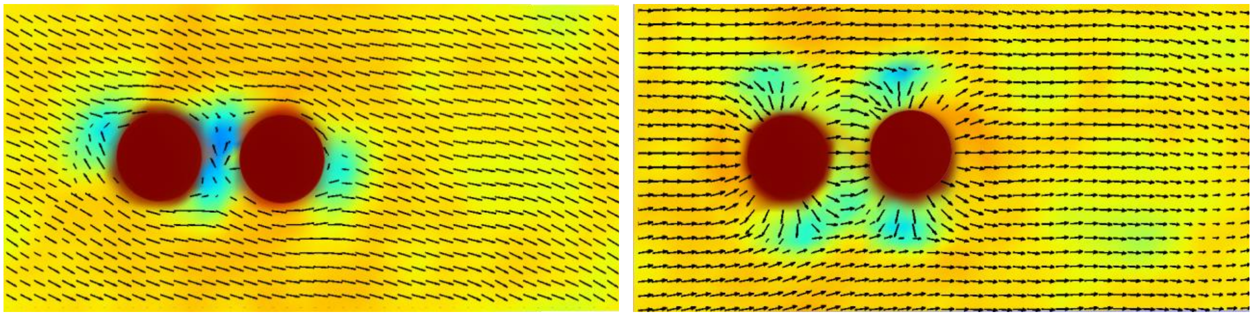

Figure S1: Visualization of the local-field director. Shown are results for the PARA case of the colloidal-pair systems, for planar [when $\kappa'' = 0.1$, (*a*)] and homeotropic [when $\kappa'' = 10$, (*b*)] anchoring. As can be gleaned from the data, the orientation of the local field becomes reasonably uniform beyond the spatial domain of the colloidal inclusions.

Data from Fig. S1 confirm that ensemble averages for the orientation of the far-field director indeed are representative of the "bulk-like" homogeneity exhibited by systems on a larger spatial scale, beyond that of the intercolloidal domain. Other anchoring strengths, including the case when a PERP arrangement is adopted, show analogous behavior.
